# Characterization of Xanthophyll Pigments, Photosynthetic Performance, Photon Energy Dissipation, Reactive Oxygen Species Generation and Carbon Isotope Discrimination during Artemisinin-Induced Stress in *Arabidopsis thaliana*

**DOI:** 10.1371/journal.pone.0114826

**Published:** 2015-01-30

**Authors:** M. Iftikhar Hussain, Manuel J. Reigosa

**Affiliations:** 1 Department of Plant Biology and Soil Science, University of Vigo, Campus Lagoas-Marcosende, 36310—Vigo, Spain; 2 International Center for Biosaline Agriculture (ICBA), P.O. Box 14660, Dubai, U.A.E; Institute for Sustainable Plant Protection, C.N.R., ITALY

## Abstract

Artemisinin, a potent antimalarial drug, is phytotoxic to many crops and weeds. The effects of artemisinin on stress markers, including fluorescence parameters, photosystem II photochemistry, photon energy dissipation, lipid peroxidation, reactive oxygen species generation and carbon isotope discrimination in *Arabidopsis thaliana* were studied. Arabidopsis ecotype Columbia (Col-0) seedlings were grown in perlite and watered with 50% Hoagland nutrient solution. Adult plants of Arabidopsis were treated with artemisinin at 0, 40, 80, 160 μM for one week. Artemisinin, in the range 40–160 μM, decreased the fresh biomass, chl *a, b* and leaf mineral contents. Photosynthetic efficiency, yield and electron transport rate in Arabidopsis were also reduced following exposure to 80 and 160 μM artemisinin. The Φ_NPQ_ and NPQ were less than control. Artemisinin treatment caused an increase in root oxidizability and lipid peroxidation (MDA contents) of Arabidopsis. Calcium and nitrogen contents decreased after 80 and 160 μM artemisinin treatment compared to control. δ^13^C values were less negative following treatment with artemisinin as compared to the control. Artemisinin also decreased leaf protein contents in Arabidopsis. Taken together, these data suggest that artemisinin inhibits many physiological and biochemical processes in Arabidopsis.

## Introduction

The herbicidal control of weeds is a costly affair and deteriorates the quality of soil, water, animal and human health, and food [1]. This situation has stimulated the interest to identify alternative weed management strategies. Plant-derived secondary compounds have great potential in the development of environmentally safe herbicides with novel molecular sites of action [2]. Natural products are, in general, structurally more complex than synthetic herbicides and would not have been obtained by traditional synthetic approaches that tend to be limited by the cost of synthesizing the final molecule [3, 4].

Artemisinin is a sesquiterpene endoperoxide lactone that is produced in biseriate glandular trichomes present on the leaves, stems and inflorescences of *Artemisia annua* [5,6]. It is derived from sesquiterpene precursor, farnesyl diphosphate, which itself is formed by the condensation of three isopentenyl diphosphate molecules synthesized either by mevalonic acid dependent or independent pathways present in the cytosol and plastid, respectively [7]. Artemisinin (1–5 μM) decreased the dry weight, frond production, chlorophyll contents, photosynthesis and respiration in *Lemna minor* [8]. It has shown to inhibit seedling growth in a number of mono and dicotyledonous plants [9]. It has also been reported as an allelopathic agent in many cases with high levels of phytotoxic activity [10–15]. Jessing et al. [16] found that EC_50_ values for the freshwater algae *Pseudokirchneriella subcapitata* and *L*. *minor* was 0.24 and 0.19 mg/L, respectively, with relative growth rate as an endpoint. Duke et al. [15] reported the selective phytotoxic properties of artemisinin because it inhibits the germination of lettuce (*Lactuca sativa* L.), but not that of redroot pigweed (*Amaranthus retroflexus* L.) and pitted glory (*Ipomoea lacunosa* L.).

Elucidating the structure and mode of actions of previously unknown natural phytotoxins could lead to new biorational approaches to weed control [17]. Several studies have set out to identify the molecular target sites of this secondary metabolite as well as its structural requirements for herbicidal activity [12, 15]. However, no definite target site has yet been identified. Inhibition of mitosis, abnormal mitotic configurations and mitotic stages suggest that artemisinin disrupts the formation of microtubule organizing centres including the loss of membrane integrity [18].

Allelopathy plays an important role in agroecosystems and offers the potential for selective biological weed management by the production and release of allelochemicals from leaves, flowers, seeds and roots of living or decomposing plant materials [19]. Artemisinin are present in various plant organs, including leaves, stems, floral and fruit parts [20]. However, there are large differences found in artemisinin content, depending on the variety, season, cultivation condition, and plant developmental stages [21. 22]. Previously, it was shown that artemisinin has potential to inhibit the seed germination of different plant species [13, 14, 15]; although no detailed research has been conducted regarding artemisinin impact on the growth of surrounding plants, or physiological and biochemical metabolism. Lydon et al. [14] found that the incorporation of *Artemisia annua* dried leaves in the soil provided good weed control, but the level of phytotoxic activity was independent of the concentrations of artemisinin in soil, suggesting that other factors may play important roles. Secondary plant metabolites play a variety of physiological roles and have a number of advantages over the synthetic herbicides as they usually have structural diversity and have novel target sites of action [23, 24]. Here, we report that artemisinin generates active oxygen species that interfere with physiological and biochemical processes in the model plant *Arabidopsis thaliana*.

## Materials and Methods

### 2.1. Plant material and growth conditions

The seeds of *Arabidopsis thaliana* L. (Heyn.) ecotype Columbia (Col-0) were surface sterilized for 3 min. in two consecutive aqueous solutions of EtOH (50%) and NaOCl (0.5%), both with Triton X-100 (0.01%), washed three times in autoclaved water; vernalized for 48 h at 4ºC in 0.1% agar to favour synchronized germination, and transferred to Petri dishes containing agar with Murashige–Skoog nutrients (Sigma–Aldrich: St. Louis, USA) and sucrose at concentrations of 1%. The Petri dishes were kept for 15 days under 60 μmol m^−2^ s^−1^ of light in a growth chamber at 22 ± 2ºC and the plantlets were transferred to individual pots, 5 cm in diameter and 6 cm high, containing inert perlite, moistened with 50% Hoagland nutrient solution (pH 6.0). These pots were placed in a growth chamber with an 8-h photoperiod, at a temperature of 22 ± 2ºC, and a photosynthetic photon flux rate of 120 μmol m^−2^ s^−1^ at canopy height. The plantlets were watered twice a week with 50% Hoagland nutrient solution containing (KNO_3_ (102 g/L), Ca (NO_3_)_2_ (50 g/L), MgSO_4_. (49 g/L), NH_4_)_2_ HPO_4_ (23 g/L), micronutrients: H_3_BO_3_ (2.86 mg/L), MnCl_2_ (2.85 mg/L), CuSO_4_ (0.08 mg/L), ZnSO_4_ (0.4 mg/L), H_2_Mo_4_ (0.02 mg/L), FeSO_4_ (2.8 g/L), Fe-ethylenediaminetetraacetic acid: Na_2_EDTA (3.72 g/L) during the first two weeks and then watered every other day until the age of 5 weeks (three more weeks in pots).

### 2.2. Artemisinin solution preparation and experimental design

Stock solution of artemisinin was prepared by dissolving in acetone. The distilled water (distilled water + tween 20 (1L/0.1 mL) was added equal to the volume of acetone, and the solution was magnetically stirred and acetone was allowed to evaporate in a rotary evaporator (Rotavapor RE 12: BUCHI Switzerland). Distilled water + tween 20 (1L/0.1 mL) was added in this solution to make a stock solution of 100 mL volume. Distilled water + tween 20 were added to this stock solution to prepare treatments having concentrations of 40, 80 and 160 μM. The procedure was repeated to prepare control without artemisinin. The pH of all these chemical solutions including control was adjusted to 6.0 with KOH. The experiment was initiated (day 0) when plants had an average of nine fully developed leaves (5 week- old). Plants were watered every other day (0, 2, 4, 6) with 15 mL of artemisinin (0, 40, 80 or 160 μM) (dissolved in 50% Hoagland nutrient solution) and physiological measurements were recorded. The plants were harvested and samples were collected for biochemical analysis. The experiment was arranged in Randomized Complete Block Design (RCBD) with four replications.

### 2.3. Chlorophyll fluorescence measurements

The fluorescence measurements were made (from day 0 up till day 7) by using a Maxi Imaging PAM Chlorophyll Fluorescence System by Walz (Effeltrich, Germany). At each measuring time, the plants were kept in darkness for 5 min to allow all reaction centres to open and minimize fluorescence associated with the energization of the thylakoid membrane, after which the whole plants were successively illuminated at an intensity of 0.5 μmol m^−2^ s^−1^ for the measurement of *F*_o_ (the minimum fluorescence of dark-adapted leaves). The leaves were illuminated with a saturating pulse of intensity 2700 μmol m^−2 −1^ for measurement of *F*_m_ (maximum fluorescence of dark-adapted leaves) and calculation of *F*_v_ = *F*_m_ − *F*_o_ and *F*_v_/*F*_m_ (maximum quantum efficiency of dark-adapted PSII) for 5 min, during which actinic illumination at 110 μmol m^−2^ s^−1^ were interrupted every 20 s with 800 ms saturating pulses of 2700 μmol m^−2^ s^−1^ for measurement of Φ_II_ (effective photochemical quantum yield, or operating efficiency, at PSII), Φ_NPQ_ (quantum yield of regulated non-fluorescent non-photochemical de-excitation), Φ_NO_ (quantum yield of all fluxes other than Φ_NPQ_ and Φ_II_, including fluorescence), *q*_N_ (non-photochemical quenching coefficient, i.e. the fraction of dark-adapted variable fluorescence that is lost upon adaptation to light); qP (photochemical quenching coefficient); *q*_L_ (fraction of PSII centres that are open), *F*′_v_/*F*′_m_ (maximum efficiency of PSII after adaptation to light) and ETR (apparent electron transport rate). All parameters were calculated as defined previously [25, 26]. Fifteen measurements were obtained for each parameter, which yielded a kinetic plot for each parameter. The integral values of the area were obtained for all of these graphs. The value represented in the graphs of fluorescence is the average area that was calculated from kinetic measurement for four replicates in each treatment. This area highlights the magnitude of change and could be used to observe the total value of the trend.

### 2.4. Chlorophyll *a*, and *b* contents

For each replicate, fresh leaves (100 mg) were homogenized in 1.5 mL of methanol and mixture was centrifuged at 170 × *g* for 5 min. A 500 μL sample of the supernatant was mixed with 500 μL of methanol and absorbance was measured on Schimadzu UV-260 Spectrophotometer at 470, 653, 666, 750 nm. Four replications were measured from each treatment. The amount of the pigments was calculated according to the simultaneous equations of Wellburn [27] as follows:

$$\text{Chl }a=\left(15.65{\text{ A}}_{666}–{\text{ A}}_{750}\right)–7.34{\text{ A}}_{663}–{\text{ A}}_{750}).\text{ V}$$ Eqn1

$$\text{Chl }b=(27.07\left({\text{A}}_{663}–{\text{ A}}_{750}\right)–11.21\left({\text{A}}_{666}–{\text{ A}}_{750}\right).\text{ V }$$ Eqn2

### 2.5. *A. thaliana* biomass determination and elemental analysis

At harvest, the C, H, and N contents of dried leaf (3 mg) were determined in a Fisons Instruments EA1108 apparatus with a detection limit of 10 ppm. The dry leaf (50 mg) was used to measure Ca^2+^, Mg^2+^, Cu^2+^, Zn^2+^, Al^3+^, Fe^2+^, Na^+^, K^+^ and PO_4_^−^ contents by inductively coupled plasma optical emission spectrometry in a Perkin Elmer Optima 4300DV.

### 2.6. Carbon isotope composition analysis

Collected plant leaf samples were immediately dried in a forced-air oven at 70ºC (Gallenkamp oven, Loughborough, Leicestershire, UK) to constant weight and ground in Ball Mills (Retsch MM 2000, Haan, Germany). Dry ground plant material were weighed (1700–2100 μg) with weighing meter (Metler Toledo GmbH: Greifensee Switzerland) and filled in tin capsules (5x3.5 mm, Elemental Microanalysis Limited, U.K.). Each tin capsule was inserted automatically into a combustion oven at 1600–1800ºC in the presence of oxygen and converted to CO_2_ and N_2_. Subsequently, isotope ratios were determined in an Isotopic Ratio Mass Spectrometer (Finnegan: Thermo Fisher Scientific, model MAT-253, Swerte Germany) coupled with an Elemental Analyzer (Flash EA-1112, Swerte Germany). The Isotopic ratio mass spectrometer has an analytical precision better than 0.3‰ for ^13^C. All preparations of isotopic analysis were performed at CACATI (Centro de Apoio Cientifico Tecnologico a la Investigacion), University of Vigo, Spain.

Carbon Isotope composition ratios are expressed in ‰ as δ values where;

$$\delta \left(‰\right)=(({\text{R}}_{\text{sample}}{\text{/R}}_{\text{standard}})—1))\text{ x }1000$$ Eqn3

Where R_sample_ is the ratio of ^13^C/^12^C and R_standard_ are standards used. Vienna PeeDee Belemnite (VPDB) was used as standard for the carbon. The accuracy and reproducibility of the measurements of δ^13^C were checked with an internal reference material (NBS 18 and IAEA-C6 for C).

Carbon isotope discrimination is a measure of the carbon isotopic composition in plant material relative to the value of the same ratio in the air on which plant feed:

Δ(‰)=((δa –δp)/(1+δp)) x 1000 Eqn4

where Δ represents carbon isotope discrimination, δa represents C isotope composition in the source air, and δp represents C isotope composition in the plant tissue. The theory published by Farquhar et al. [28] and Farquhar & Richards [29] indicates that carbon discrimination in leaves of plants can be expressed in relationship to CO_2_ concentrations inside and outside of leaves in its simplest form as:

$$\begin{array}{l} \Delta =a+\left(b-a\right)c\text{i}/c\text{a}\\ \Delta =4.4+\left(27-4.4\right)c\text{i}/c\text{a} \end{array}$$ Eqn5

where *a* is discrimination that occurs during diffusion of CO_2_ through the stomata (4.4‰), *b* is discrimination by Rubisco (27‰), and *c*i/*c*a is the ratio of the leaf intercellular CO_2_ concentrations to that in the atmosphere. Equation (3) shows a direct and linear relationship between Δ and *c*i/*c*a. Therefore, measurement of Δ will provide an estimation of the assimilation-rate—weighted value of *c*i/*c*a. Data of δ ^13^C_air_, *c*i and *c*a were obtained from McCarroll & Loader [30] who used the high precision records of atmospheric Δ^13^C from Antarctic ice cores, and the atmospheric CO_2_ concentrations (ppm) from Robertson et al. [31].

### 2.7. Root oxidizability

Root oxidizability is an indirect estimation of tissue viability, and is determined using 2,3,5-triphenyl tetrazolium chloride (TTC) [32]. Viable (i.e., respiring) tissue reduces TTC to red coloured triphenyl formazan by accepting electrons from mitochondrial electron transport chain. Thus, any decrease in root oxidizability refers to reduced respiration resulting from tissue damage and, therefore, reduced viability. Briefly, root tissue (50 mg) was treated with 5 mL of 0.4% TTC solution (w/v) and 5 mL of 1/15 M potassium phosphate buffer (pH 7.4). The mixture was incubated at 40ºC for 3 h followed by the addition of 2 mL 2.0 *N* H_2_SO_4_. Thereafter, the roots were ground in 10 mL of reagent grade ethyl acetate to extract red triphenyl formazan that was recorded at 485 nm and expressed as A_485_ g^–1^ h^–1^.

### 2.8. Lipid peroxidation

At harvest, lipid peroxidation was determined by measurement of malonyldialdehyde (MDA) content [33]. Pre-frozen plant material (120 mg) were homogenized in 80% ethanol and centrifuged at 3000 × *g* for 10 min at 4ºC, the supernatant was incubated at 95ºC with 20% TCA containing 0.01% hydroxytoluenebutylate, with and without 0.5% thiobarbituric acid (TBA). Absorbance was measured at 440, 532 and 600 nm. The lipid peroxidation was calculated as the malonyldialdehyde (MDA) (nmol/mL) equivalents according to this equation;

$$\text{MDA }\left(\text{nmol/mL}\right)=((\text{A – B})/157)\text{ x }10^{3}$$ Eqn6

where $A={\left({\text{Abs}}_{532}-{\text{Abs}}_{600}\right)}_{\text{TBA}+}-{\left({\text{Abs}}_{532}-{\text{Abs}}_{600}\right)}_{\text{TBA}-}$and $B=0.0571\times {\left({\text{Abs}}_{440}-{\text{Abs}}_{600}\right)}_{\text{TBA}+}.$

### 2.9. Leaf protein content

After harvest total protein content was determined by Bradford's method [34]. For each replicate, fresh leaves (100 mg) were homogenized in 0.8 mL of 0.05 M Tris buffer (pH 7.4) containing 0.05 g of the antioxidant polyvinyl polypyrrolidone, and the mixture was centrifuged at 2860 *g* for 20 min at 4ºC. A 0.1 mL sample of the supernatant was mixed with Bradford's reagent, and absorbance at 595 nm was measured and translated into protein content using a calibration curve constructed with bovine serum albumin as standard.

### 2.10. Statistical analysis

Following testing for non-normality by the Kolmogorov—Smirnov test and Levene’s testing for heteroscedasticity, the significance of differences among group means was estimated by analysis of variance followed by the Duncan test for homoscedastic data or by the Dunnett test for heteroscedastic data using SPSS 17.0 statistical package (SPSS, Chicago, IL, USA). Each value represents the mean (± S.E.) of four replicates.

## Results

Previously, we reported the weed suppressive potential of artemisinin on germination and seedling growth of *Arabidopsis thaliana* in a laboratory setting bioassay [35]. This experiment was undertaken to evaluate the physiological, biochemical and isotopic responses of *A*. *thaliana* under allelopathic stress of artemisinin.

### 3.1. Effect of artemisinin on leaf biomass of *A*. *thaliana*

Exposure of Arabidopsis to artemisinin caused a dose-dependent reduction in fresh weight (Table 1), although maximum decrease was observed at 160 μM artemisinin. At 80 μM, artemisinin, caused 52% reduction in fresh biomass while exposure of the Arabidopsis leaves to 160 μM artemisinin exacerbated the phytotoxic effects and fresh biomass in these plants represented 59% less than the control plants (Table 1) and formation of necrosis on the outer surface of the leaf zone (personal observation). Artemisinin decreased the leaf dry mass at 160 μM as compared to control. However, there were no differences in leaf dry weight between control and treated plants at all other concentrations. Furthermore, artemisinin reduced the leaf fresh/dry weight ratio at 80 and 160 μM concentrations (Table 1). The leaf carbon (C) and nitrogen (N) contents in Arabidopsis decreased after treatment with 160 μM artemisinin. Artemisinin (40, 80 and 160 μM) increased the sodium, potassium, phosphorus and aluminium contents in Arabidopsis leaves, following 7 days exposure (Table 2). Simultaneous with the above changes in sodium, potassium and phosphorus, no reduction of hydrogen and copper contents in Arabidopsis leaves was observed following artemisinin treatment (Table 3).

**Table 1:** Effects of Artemisinin (0, 40, 80, 160 μM) on leaf fresh and dry weight (g) and leaf fresh/dry weight ratio of *Arabidopsis thaliana*.

Artemisinin			
(μM concentrations)	Fresh weight (FW) (g)	Dry weight (DW) (g)	FW/DW Ratio
0 (Control)	0.363±0.05a	0.048±0.01a	11.73±3.83a
40	0.310±0.06a	0.042±0.01a	10.77±3.59a
80	0.175±0.03b	0.042±0.007a	4.92±1.58b
160	0.150±0.007c	0.035±0.009b	5.44±1.48b

Means followed by different letters are significantly different at *p* < 0.005. Kolmogorov—Smirnov testing for non-normality and Levene’s testing for heteroscedasticity. Statistical significance of differences among group means was estimated by analysis of variance followed by Duncan test for homoscedastic data and by the Dunnett test for heteroscedastic data. Each value represents the mean (± S.E.) of four replicates.

**Table 2:** Carbon (%) and nitrogen (%) and C/N ratio in leaves of thale cress (*Arabidopsis thaliana*) following 1 week exposure to artemisinin at 0, 40, 80, 160 μM concentrations.

Artemisinin			
(μM concentrations)	Carbon (%)	Nitrogen (%)	C/N Ratio
0 (Control)	34.55±0.19a	7.32±0.09a	4.72±0.07a
40	34.05±0.41a	7.48±0.10a	4.55±0.11a
80	33.72±0.65a	7.38±0.19a	4.58±0.21a
160	28.84±0.28b	6.29±0.10b	4.58±0.05a

Means followed by different letters are significantly different at *p* < 0.005. Kolmogorov—Smirnov testing for non-normality and Levene’s testing for heteroscedasticity the statistical significance of differences among group means was estimated by analysis of variance followed by Duncan test for homoscedastic data and by Dunnett test for heteroscedastic data. Each value represents the mean (± S.E.) of four replicates.

**Table 3:** Sodium (mg g^−1^), potassium (mg g^−1^), phosphate (mg g^−1^), hydrogen (mg g^−1^), aluminium (mg kg^−1^) and copper (mg kg^−1^) contents in leaves of thale cress following 1 week exposure to artemisinin at 0, 40, 80, 160 μM concentrations in leaves of *Arabidopsis thaliana*.

Artemisinin	Sodium	Potassium	Phosphate	Hydrogen	Aluminium	Copper
(μM concentrations)	(mg g^−1^)	(mg g^−1^)	(mg g^−1^)	(mg g^−1^)	(mg kg^−1^)	(mg kg^−1^)
0 (Control)	8552.72± 45a	56085.77±69a	13499.95±579a	5.25±0.05a	272.14±24.92a	9.19±0.12a
40	9519.27±59b	62329.35±81b	15220.34±614b	5.08±0.06a	359.51±90.96b	9.26±0.79a
80	9100.78±10b	60273.83±24b	15144.49±429b	5.15±0.06a	565.61±176.88b	9.06±0.36a
160	11061.27±68b	65546.46±15b	16858.75±340b	5.03±0.02a	401.99±72.74b	9.33±0.23a

Means followed by different letters are significantly different at *p* < 0.005. Kolmogorov—Smirnov testing for non-normality and Levene’s testing for heteroscedasticity. Statistical significance of differences among group means was estimated by analysis of variance followed by Duncan test for homoscedastic data and by the Dunnett test for heteroscedastic data. Each value represents the mean (± S.E.) of four replicates.

### 3.2. Effect of artemisinin on chlorophyll pigments

**Figure 1:**
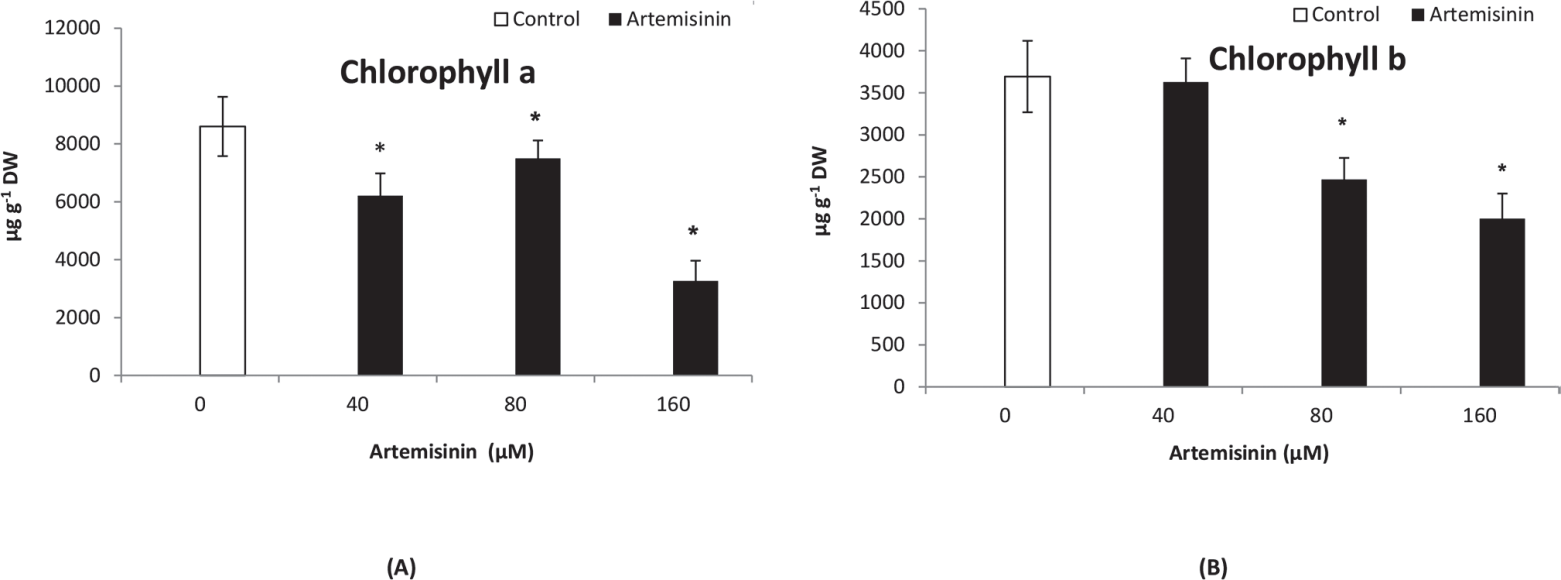
Photosynthetic pigments, chlorophyll *a* (A) and *b* (B) in Arabidopsis leaves after treatment with 0, 40, 80, and 160 μM artemisinin and untreated control. Whole plants were measured and the values integrated afterwards. Fifteen measures were obtained for each parameter at each measuring time, which gave a kinetic plot for each parameter along the time. The integral value of the area was obtained for each parameter at every time. *Asterisks show the statistical significance as compared to control at *p* < 0.005. After Kolmogorov—Smirnov testing for non-normality and Levene’s testing for heteroscedasticity, the statistical significance of differences among group means was estimated by analysis of variance followed by Duncan test for homoscedastic data and by Dunnett test for heteroscedastic data.

Chlorophyll *a* and *b* contents decreased at 40, 80 and 160 μM artemisinin. The chl *a* content decreased by approximately 40% compared to the control following exposure to 160 μM artemisinin (Fig. 1A). The chl *b* contents in Arabidopsis leaves were reduced after treatment with 80 and 160 μM artemisinin and the reduction was 20% and 15%, in terms of the control (Fig. 1B).

### 3.3. Artemisinin dosage-inhibiting curves of F_v_/F_m_, Φ_II_ and ETR

**Figure 2:**
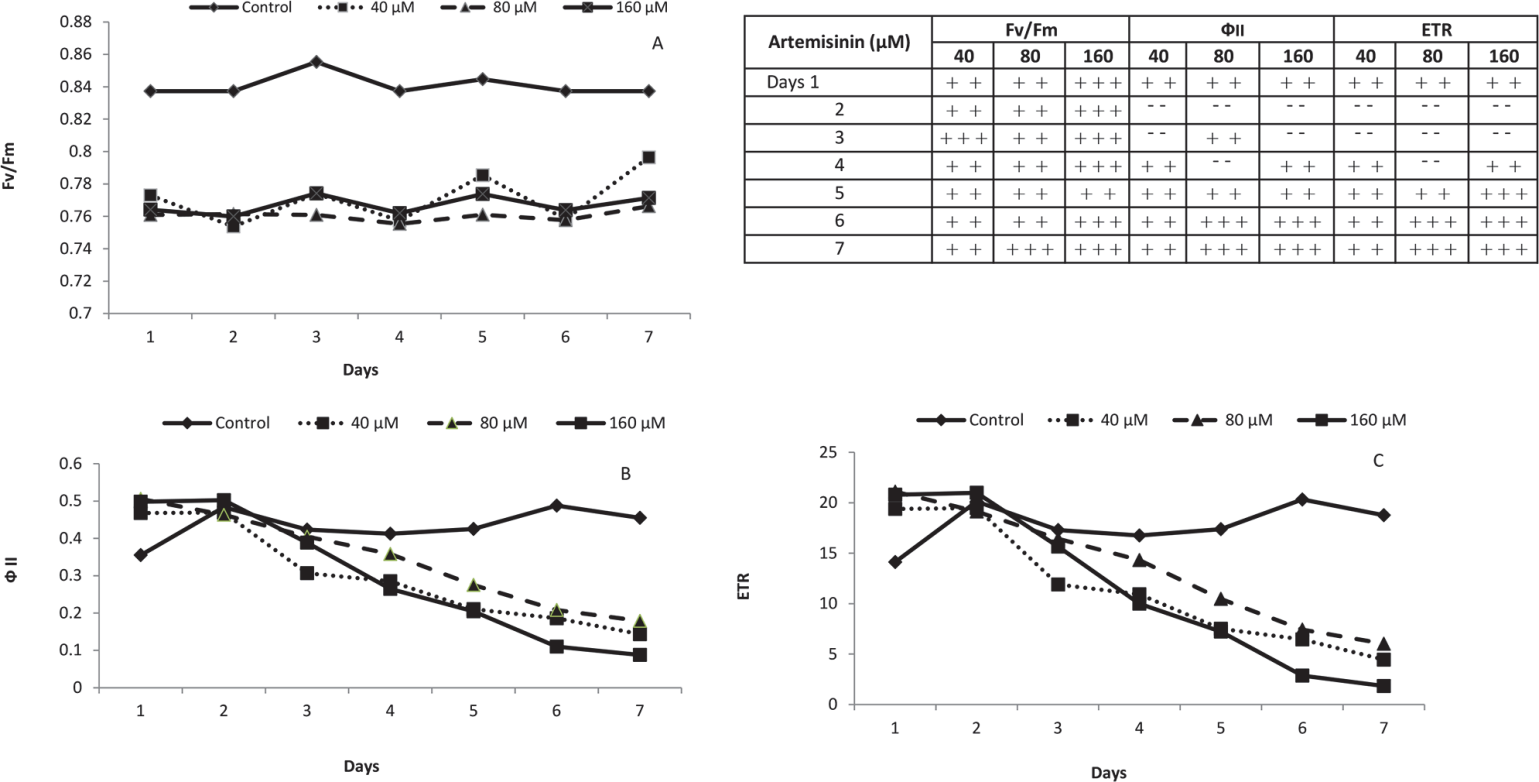
Values of maximum photosynthetic efficiency of dark-adapted PSII (F_v_/F_m_) (A), maximum photosynthetic yield (Ф II) (B) and apparent electron transport rate (ETR) (C) in whole Arabidopsis plants after treatment with 0, 40, 80 and 160 μM artemisinin. Whole plants were measured and the values integrated afterwards. Fifteen measures were obtained for each parameter at each measuring time, which gave a kinetic plot for each parameter along the time. The integral value of the area was obtained for each parameter at every time. The table shows the statistical significance of positive (+) or negative (-) differences with respect to untreated plants. Plus signs indicate positive differences with respect to controls and minus signs negative differences. The number of plus or minus signs indicates statistical significance: one, *p* < 0.05; two, *p* < 0.01; three, *p* < 0.001.. After Kolmogorov—Smirnov testing for non-normality and Levene’s testing for heteroscedasticity, the statistical significance of differences among group means was estimated by analysis of variance followed by Duncan test for homoscedastic data and by Dunnett test for heteroscedastic data.

Artemisinin treatments reduced the photosynthetic efficiency under the dark adapted state (F_v_/F_m_) in *A*. *thaliana* up till day 7 at 40, 80 and 160 μM compared to the control (Fig. 2A). Artemisinin reduced the photosynthetic yield Φ_II_ during all 7 days after artemisinin treatment at 40 μM, but it was on a par with control on day 2 and 3 while, once again, it decreased on day 4 till day 7 in terms of the control (Fig. 2B). Meanwhile, at 80 μM there was an uneven effect during the first 4 days, but however, during day 5, Φ_II_ values decreased up to day 7, in terms of the control. The artemisinin at 40, 80 and 160 μM decreased the electron transport rate (ETR) during day 1, but however, it was on a par with control on day 2 and 3. From day 4 – 7 artemisinin decreased ETR values at all concentrations in terms of the control. Meanwhile, at 80 and 160 μM artemisinin, there was a strong inhibition of ETR values during day 6 and 7 in terms of the control (Fig. 2C).

### 3.4. Effect of artemisinin on Φ_NPQ,_ NPQ and Φ(NO)

**Figure 3:**
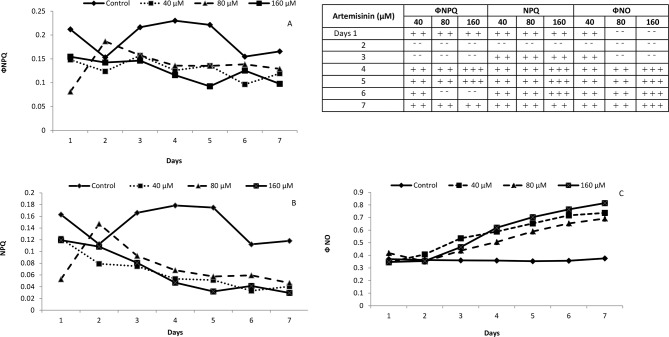
Values of the quantum yield of light-induced non-photochemical quenching (NPQ) quantum yield of all photosynthetically active photon fluxes other than ФNPQ, NPQ and ФNO in whole Arabidopsis plants after treatment with 0, 40, 80 and 160 μM artemisinin. Whole plants were measured and the values integrated afterwards. Fifteen measures were obtained for each parameter at each measuring time, which gave a kinetic plot for each parameter along the time. The integral value of the area was obtained for each parameter at every time. Table shows the statistical significance of positive (+) or negative (-) differences with respect to untreated plants. Plus signs indicate positive differences with respect to controls and minus signs negative differences. The number of plus or minus signs indicates statistical significance: one, *p* < 0.05; two, *p* < 0.01; three, *p* < 0.001. After Kolmogorov—Smirnov testing for non-normality and Levene’s testing for heteroscedasticity, the statistical significance of differences among group means was estimated by analysis of variance followed by Duncan test for homoscedastic data and by Dunnett test for heteroscedastic data.

Excitation energy fluxes in these three different pathways could easily be assessed by imaging-PAM chlorophyll fluorometer. Three fluxes recorded every day of artemisinin application confirmed the differential artemisinin inhibitory impact on Arabidopsis. Artemisinin at all concentrations (40, 80, 160 μM) decreased values of Φ_NPQ,_ NPQ and but Φ (NO), in Arabidopsis was only less than control following treatment at 40 and 160 μM artemisinin from day 1 – 3 (Fig. 3 A, B, C). The reduction of NPQ in Arabidopsis leaves was detected at all concentrations of artemisinin from day 4 to day 7 (Fig. 3B), whereas Φ_NPQ_ were decreased following treatment with 40, 80, 160 μM artemisinin from day 4 up till day 7 (Fig. 3A). The data exhibited typical saturation kinetics and also revealed inter-specific differences in heat energy dissipation in *A*. *thaliana* during exposure to increased artemisinin levels. However, the inhibition of NPQ became more serious in *A*. *thaliana* after treatment at 40, 80 and 160 μM during the last 5 days. At the same time, inhibition of Φ_NPQ_ was detected in Arabidopsis following treatment at all artemisinin concentrations. However, there was a tendency towards stimulation in Φ (NO) in *A*. *thaliana* from day 4 to 7 (Fig. 3C). The above results showed that the excitation energy fluxes of NPQ and Φ_NPQ_ can be used as the biomarkers for rapid phytotoxicity assessment.

### 3.5. Artemisinin dosage-inhibiting effect on q_N,_ q_P_ and q_L_

**Figure 4:**
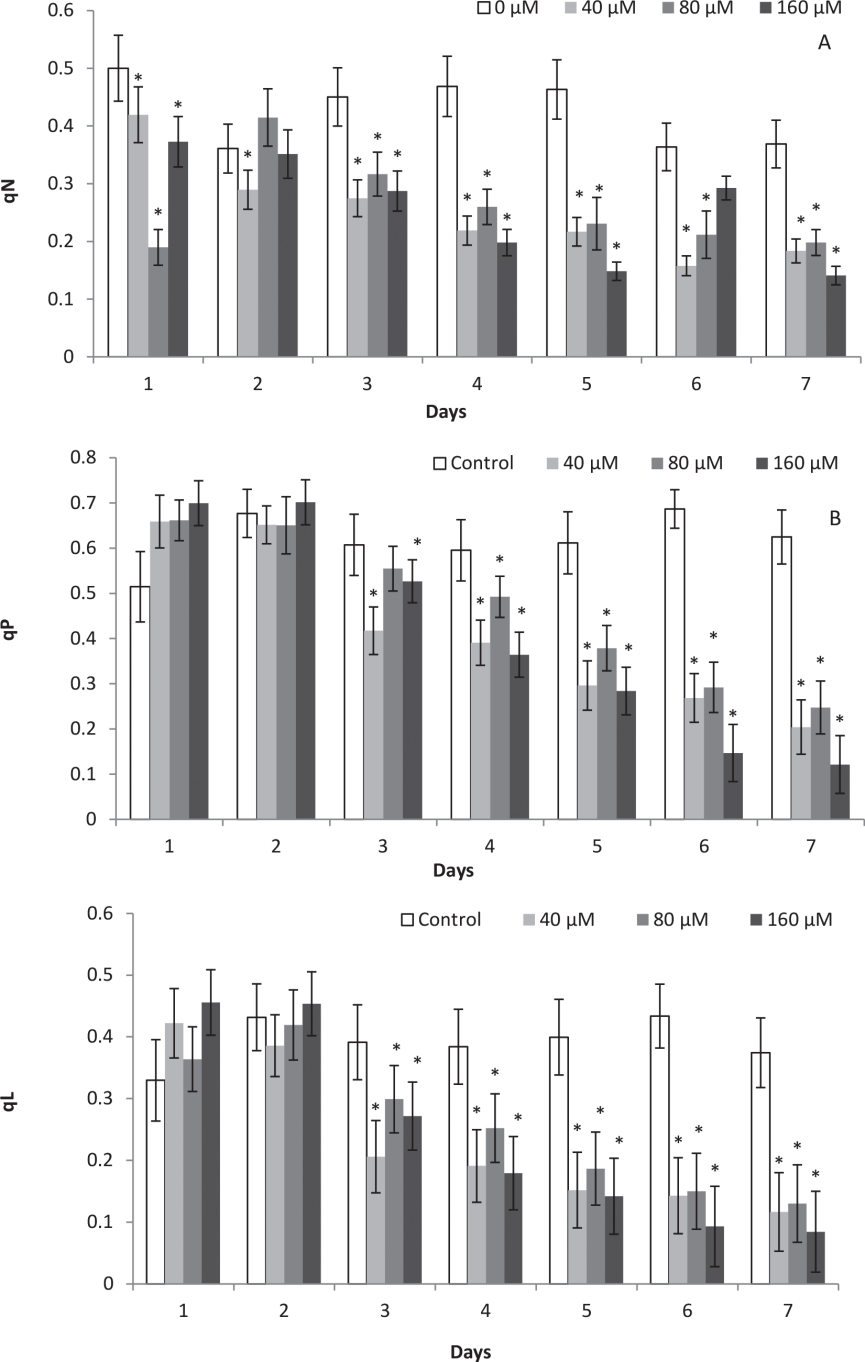
Values of the chlorophyll fluorescence quenching coefficients (qP, qL, qN) in whole Arabidopsis plants after treatment with 0, 40, 80, 160 μM artemisinin. Whole plants were measured and the values integrated afterwards. Fifteen measures were obtained for each parameter at each measuring time, which gave a kinetic plot for each parameter along the time. The integral value of the area was obtained for each parameter at every time. *Asterisks show the statistical significance as compared to control at *p* < 0.005. After Kolmogorov—Smirnov testing for non-normality and Levene’s testing for heteroscedasticity, the statistical significance of differences among group means was estimated by analysis of variance followed by Duncan test for homoscedastic data and by Dunnett test for heteroscedastic data.

Artemisinin at 40, 80 and 160 μM declined *q*_N_ in Arabidopsis during all 7 days (Fig. 4A). The values of *q*_P_ decreased following treatment with artemisinin from day 3 to 7 than control at all concentrations. There was a stronger inhibition in values of *q*_P_ during last three days and 50% reduced in terms of control after artemisinin exposure at 160 μM (Fig. 4B). The artemisinin reduced the *q*_L_ (the fraction of PSII centres that are open) from day 3 to day 7 following exposure to artemisinin at 40, 80, 160 μM. However, there was no significant effect on *q*_L_ during the first two days at all artemisinin concentrations in terms of the control (Fig. 4). From day 4 to 7, there was severe damage to non-photochemical quenching coefficient (*q*_L_) at all concentrations tested (Fig. 4C). The changes of artemisinin dosage-inhibiting effect on *q*_P_, *q*_N_ and *q*_L_ in Arabidopsis showed a different trend i.e., there was a tendency to increase in values of *q*_N_ and *q*_L_ during the initial two days following artemisinin treatment.

### 3.6. Effects of artemisinin on root oxidizability and MDA content

**Figure 5:**
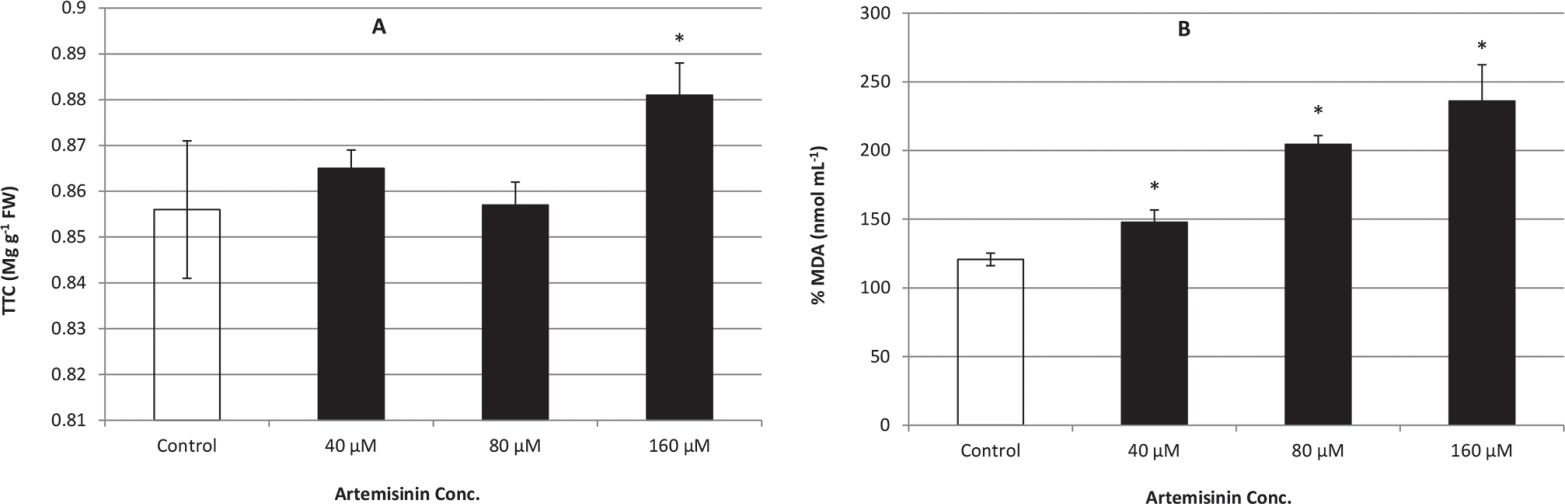
Changes in leaf lipid peroxidation (% malondialdehyde content nmol/mL) and root oxidizability (TTC mg/g FW) in *A*. *thaliana* following treatment with artemisinin (0, 40, 80, 160 μM). *Asterisks show the significant difference at *p* < 0.005. Kolmogorov—Smirnov testing for non-normality and Levene’s testing for heteroscedasticity. The statistical significance of differences among group means was estimated by analysis of variance followed by Duncan test for homoscedastic data and by Dunnett test for heteroscedastic data. Each value represents the mean (± S.E.) of four replicates.

The root oxidizability was measured in terms of triphenyltetrazolium chloride in Arabidopsis roots. Artemisinin stress caused an increase in root oxidizability (RO) in Arabidopsis at highest concentrations. Increasing the artemisinin concentrations in treated pots caused a gradual increase in the RO in Arabidopsis roots (Fig. 5 A). At 160 μM artemisinin, the tendency was to increase RO, which was highest in terms of the control.

There was a concentration dependent increase in lipid peroxidation following artemisinin exposure (Fig. 5 B). After exposure to 40 and 80 μM artemisinin, the level of MDA increased corresponding to 1.85 and 1.56 times higher than control, respectively. At 160 μM artemisinin, MDA content increased by 31% over the control (Fig. 5 B). Increased RO along with increased MDA content indicated the occurrence of artemisinin-induced stress in Arabidopsis.

### 3.7. Effects of artemisinin on Arabidopsis leaf nutrient content

**Figure 6:**
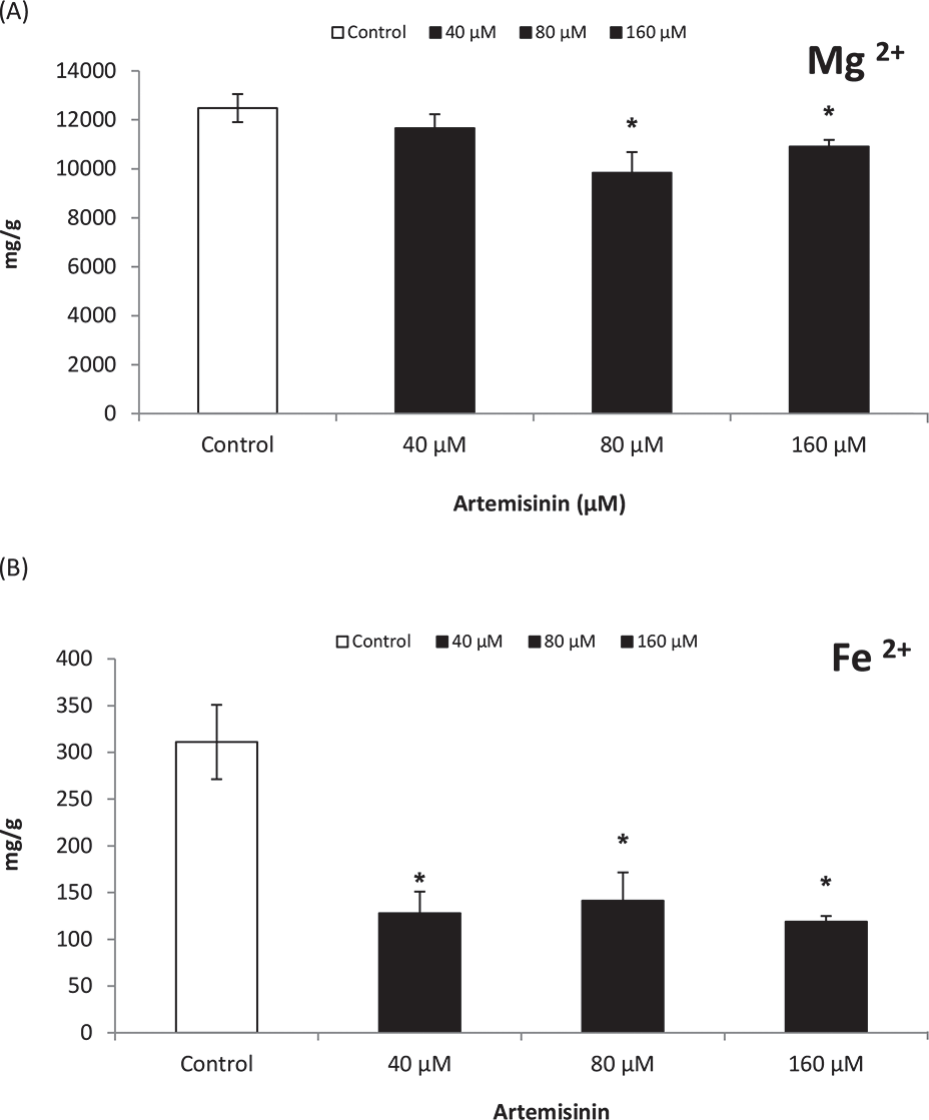
Magnesium and iron contents (dry weight basis) of leaves of thale cress treated with different concentrations (0, 40, 80, and 160 μM) of artemisinin and in untreated control one week after treatment. *Asterisks show the statistical significance as compared to control at *p* < 0.005. After Kolmogorov—Smirnov testing for non-normality and Levene’s testing for heteroscedasticity, the statistical significance of differences among group means was estimated by analysis of variance followed by Duncan test for homoscedastic data, and by Dunnett test for heteroscedastic data. Each value represents the mean (± S.E.) of four replicates.

**Figure 7:**
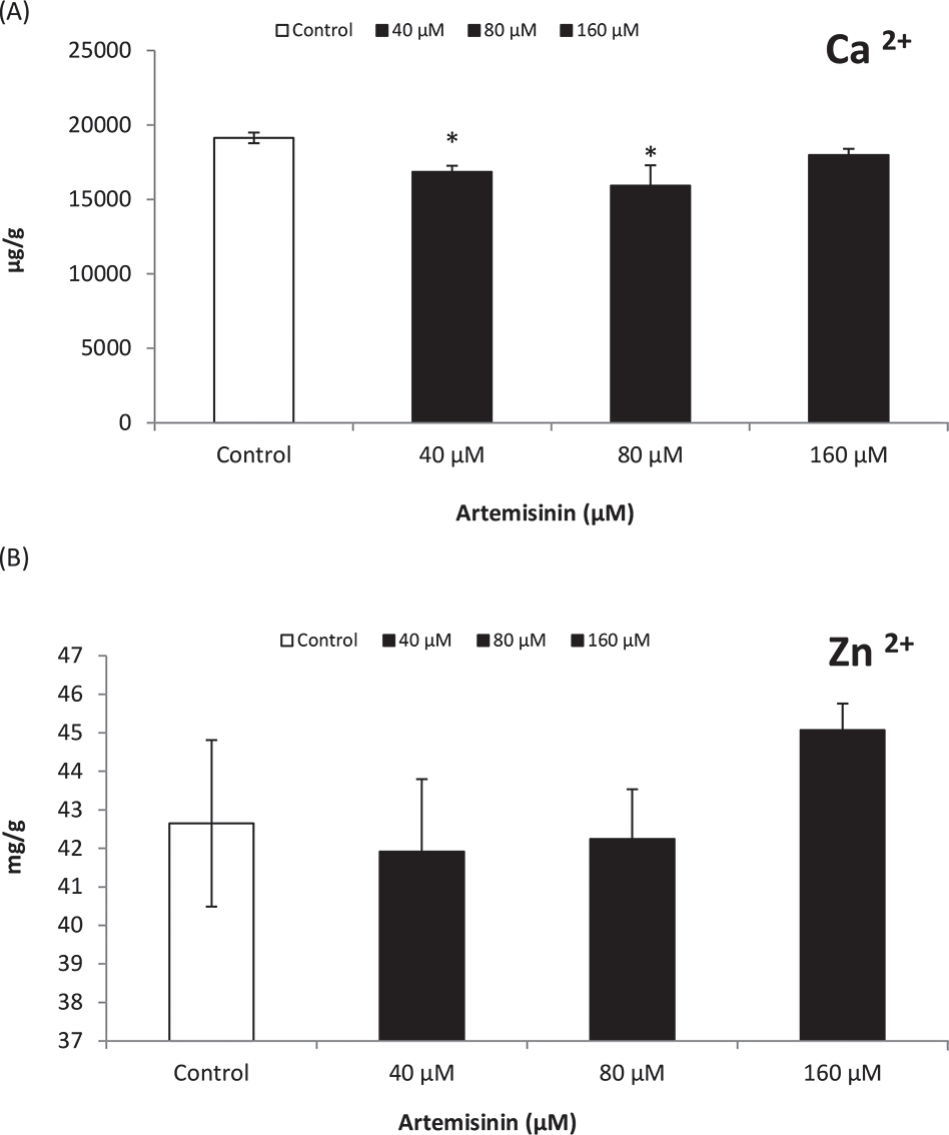
Zinc and calcium contents (dry weight basis) of leaves of *A*. *thaliana* treated with different concentrations (0, 40, 80, 160 μM) of artemisinin and in untreated control one week after treatment. *Asterisks show the statistical significance as compared to control at *p* < 0.005. After Kolmogorov—Smirnov testing for non-normality and Levene’s testing for heteroscedasticity, the statistical significance of differences among group means was estimated by analysis of variance followed by Duncan test for homoscedastic data and by Dunnett test for heteroscedastic data. Each value represents the mean (± S.E.) of four replicates.

A reduction in Mg^2+^content was observed in Arabidopsis leaves after treatment with 80 and 160 μM artemisinin (Fig. 6), while Mg^2+^ content remained unaltered at lower concentrations (40 μM). In Arabidopsis, Fe^2+^ contents decreased drastically following exposure to artemisinin at 40 and 80 μM concentrations while maximum reduction was noted at highest concentrations (160 μM) (Fig. 6). The Ca^2+^ content in Arabidopsis leaves decreased following treatment at 40 and 80 μM artemisinin concentrations (Fig. 7). There was a tendency of stimulation in Zn^2+^ content in Arabidopsis leaves after artemisinin treatment (160 μM).

### 3.8. Effect of artemisinin on Arabidopsis leaf protein contents

**Figure 8:**
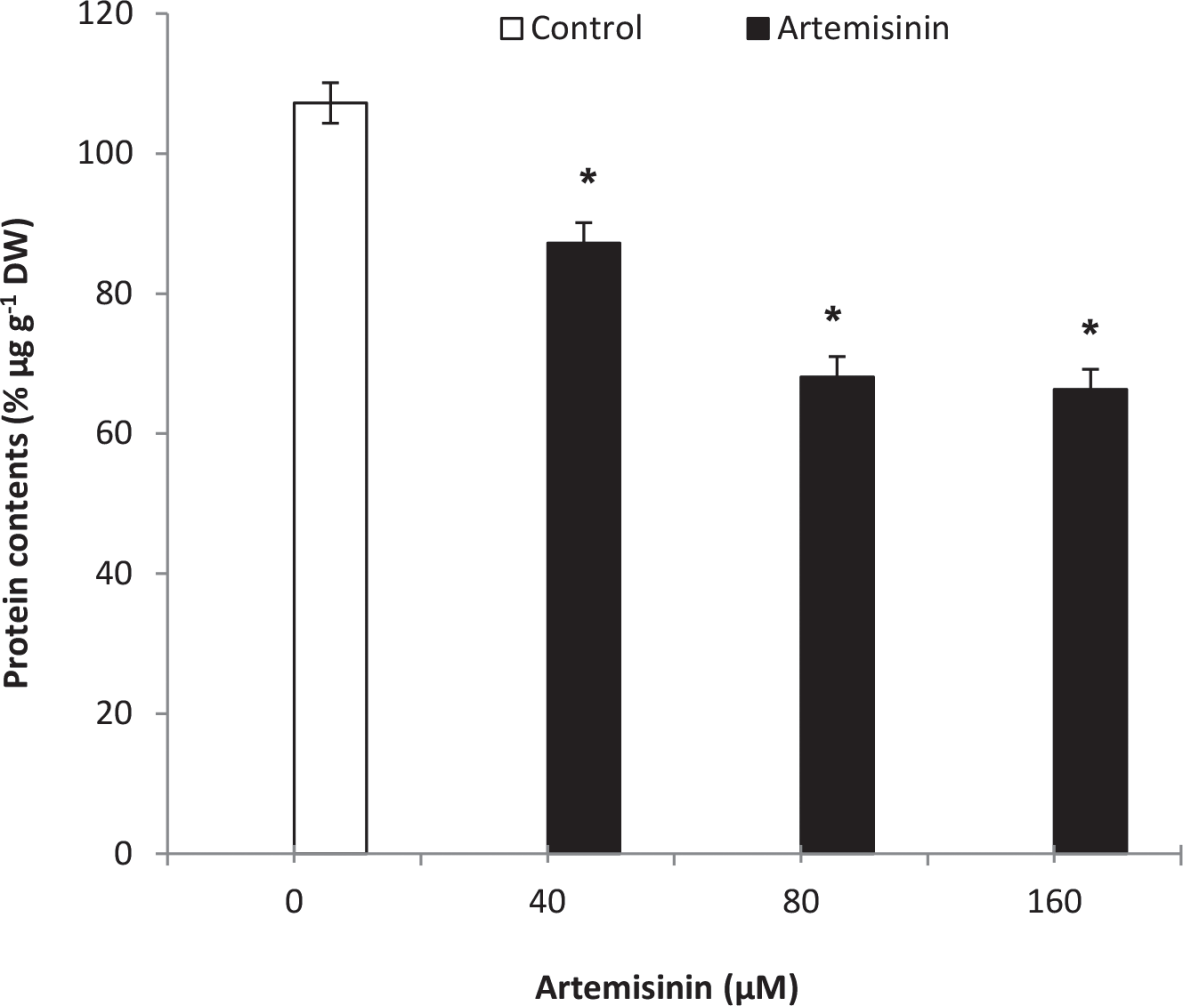
Changes in leaf protein contents in leaves of *A*. *thaliana* following exposure to one week of artemisinin at 40, 80 and 160 μM concentrations and untreated 0 (Control). *Asterisks show the statistical significance as compared to control at *p* < 0.005. After Kolmogorov—Smirnov testing for non-normality and Levene’s testing for heteroscedasticity, the statistical significance of differences among group means was estimated by analysis of variance followed by the Duncan test for homoscedastic data and by the Dunnett test for heteroscedastic data. Each value represents the mean (± S.E.) of four replicates.

Artemisinin decreased leaf protein contents of *A*. *thaliana* at all concentrations. The highest reduction (38.%) in protein contents in Arabidopsis leaves was obtained following exposure to 160 μM artemisinin as compared to the control (Fig. 8).

### 3.9. Effects of artemisinin on carbon isotope composition in Arabidopsis leaf

**Figure 9:**
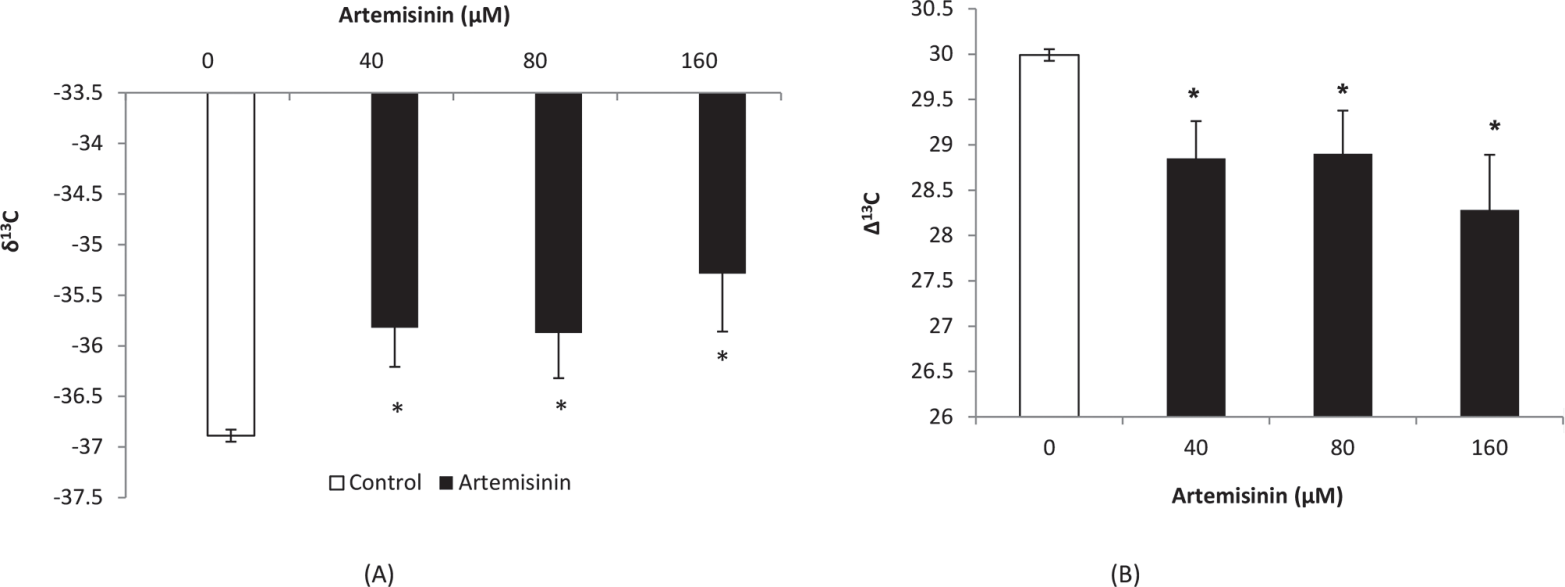
Changes in carbon isotope composition (δ^13^C) and carbon isotope discrimination (Δ^13^C) in leaves of *A*. *thaliana* following exposure to artemisinin at 0, 40, 80, 160 μM. *Asterisks show the statistical significance as compared to control at *p* < 0.005. After Kolmogorov—Smirnov testing for non-normality and Levene’s testing for heteroscedasticity, the statistical significance of differences among group means was estimated by analysis of variance followed by Duncan test for homoscedastic data and by Dunnett test for heteroscedastic data. Each value represents the mean (± S.E.) of four replicates.

**Figure 10:**
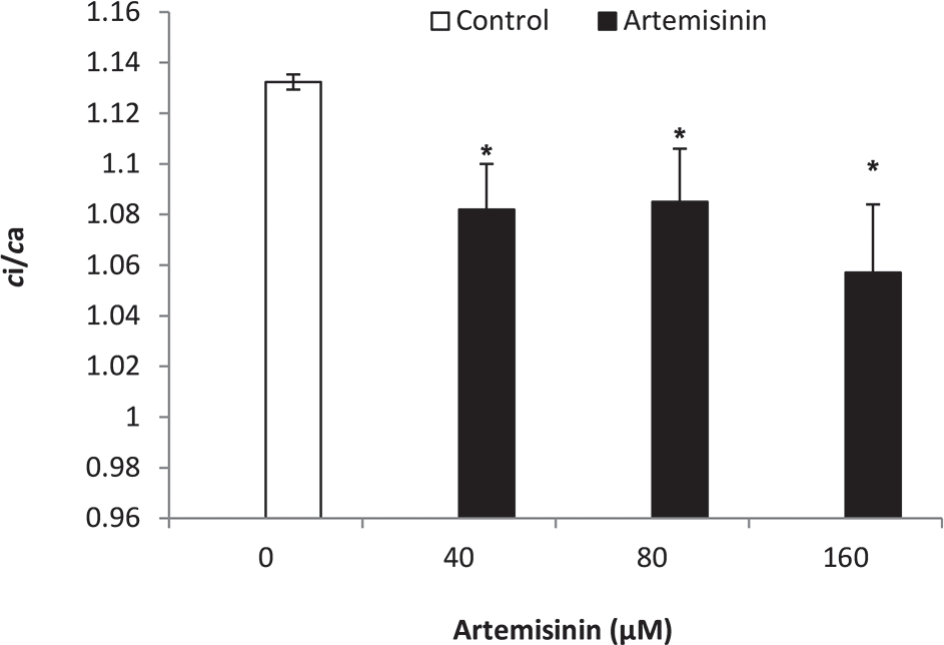
Ratio of intercellular CO_2_ concentrations from leaf to air (*c*i/*c*a) in *A*. *thaliana* following exposure to different concentrations of artemisinin at 0, 40, 80, 160 μM. *Asterisks show the statistical significance as compared to control at *p* < 0.005. After Kolmogorov—Smirnov testing for non-normality and Levene’s testing for heteroscedasticity, the statistical significance of differences among group means was estimated by analysis of variance followed by Duncan test for homoscedastic data, and by the Dunnett test for heteroscedastic data. Each value represents the mean (± S.E.) of four replicates.

In this study, the carbon isotope composition (δ^13^C) values in Arabidopsis leaves were less negative (–35.82, –35.87 and—35.28‰) following treatment with 40, 80 and 160 μM artemisinin, respectively, compared to the control (–36.88‰) (Fig. 9). The carbon isotope discrimination (Δ^13^C) values in leaves of *A*. *thaliana* were decreased (28.85, 28.90) after treatment at 40 and 80 μM artemisinin concentrations as compared to control (29.99) and the maximum reduction in Δ^13^C values (28.28) was observed at 160 μM artemisinin concentrations (Fig. 9). The Δ^13^C values indicate that limiting the diffusion of CO_2_ through the stomatal aperture can result in less negative δ^13^C values. At 40 and 80 μM concentrations, the artemisinin decreased the ratio of intercellular CO_2_ from inside to outside environment (*c*i/*c*a) while at 160 μM, there was a maximum reduction in *c*i/*c*a ratio (1.057) as compared to control (1.132) (Fig. 10).

## Discussion

Despite the pharmaceutical importance of plant-derived artemisinin, relatively little is known about the biochemical, physiological and isotopic responses to artemisinin in target plants. Here, we studied the fundamental biological processes by investigating the Arabidopsis plants’ dynamic response to phytochemical treatments, interference in growth, development and physio-biochemical characteristics. In accordance with DiTomaso & Duke [36] and Lommen et al. [37], we confirmed that artemisinin was able to decrease the fresh biomass in a dose-dependent fashion (Table 1). Chen and Polatnick [38] reported that the fresh weight of mung bean seedlings treated with artemisinin was 19–26% less as compared to control. In this experiment, the reduction in the carbon and nitrogen content of Arabidopsis leaf was observed after treatment with artemisinin at 160 μM concentration (Table 2). Lydon et al. [14] found that *Artemisia annua* leaf tissue incorporated into the soil decreased the soybean biomass in a pot culture experiment. In this study, artemisinin increased the sodium, potassium, phosphorus, aluminium and copper contents. Conversely, Sánchez-Moreiras et al. [39] found a reduction in leaf nutrient contents in Arabidopsis after treatment with BOA (2–3*H*-benzoxazolinone (BOA).

In all photosynthetic organisms, chlorophyll plays a central role in the harvesting and photochemical transformation of light energy into chemical energy. The excitation energy absorbed by the light harvesting complex (LHC) can usually undergo three fates; it can drive photosynthesis (photochemistry), be dissipated as heat or as red fluorescence. These three processes occur alongside each other. Therefore, determining the yield of chlorophyll fluorescence will give information about changes in the efficiency of photochemistry and heat dissipation [40]. Leaf photosynthetic pigment contents are good indicators for stress detection and tolerance [41]. Chl *a* and *b* contents were decreased after artemisinin treatment (Fig. 1) and this is in accordance with a correspondence decrease in F_v_/F_m_ ratio. The decrease of Chl *a* and *b* contents in Arabidopsis indicate a preferential decrease in light-harvesting chlorophyll *a*/*b*-binding proteins associated with PSII (LHCII) to transfer excitation energy to the PSII core complex [42]. The decrease in LHCII due to reduction in the light absorption cross-section of the photosystem is an essential protection mechanism, which allows plants to survive in unfavourable conditions [43]. The artemisinin stress modifies the chlorophyll *a* content more than chlorophyll *b*, which appears to be less sensitive to artemisinin stress treatment.

Artemisinin treatments reduced photosynthetic efficiency under the dark adapted state (F_v_/F_m_) in *A*. *thaliana* during all days. Other researchers also reported a variable decrease in photosynthesis, transpiration rate, stomatal conductance and water use efficiency in different plant species under allelochemical stress [44, 45]. The fall in F_v_/F_m_ indicates a loss of photosynthetic capacity, possibly due to degradation of antenna pigments or proteins. Artemisinin reduced the photosynthetic yield Φ_II_ during all 7 days. At 80 and 160 μM artemisinin, inhibited electron transport rate (ETR) in Arabidopsis from day 4 – 7 (Fig. 2). Bharati et al. [44] reported that artemisinin is converted, in vivo, to a derivative that inhibits photosynthetic electron transport. Allelochemicals appear to alter a variety of physiological processes including direct inhibition of PSII components and ion uptake, interruption of dark respiration and ATP synthesis, thylakoid electron transport (light reaction), and ROS-mediated allelopathic mechanisms [45].

To investigate the contribution of artemisinin towards the inhibition of non-photochemical fluorescence and heat energy dissipation; we describe here the phytotoxic impact of artemisinin on excitation energy fluxes; Φ_NPQ,_ NPQ and Φ (NO), namely photochemical utilization, regulated heat dissipation energy (a loss process serving for protection) and non-regulated heat dissipation energy (a loss process due to PS II inactivity), respectively. The Non-Photochemical Quenching (NPQ) of chlorophyll fluorescence is the most efficient photoprotective response in plants. As soon as the absorbed energy by light LHC exceeds the requirement for photochemical activity, this fast mechanism of heat dissipation is triggered in order to prevent ROS production [46]. The reduction of NPQ in Arabidopsis leaves was detected at all concentrations of artemisinin from day 4 – 7, whereas Φ_NPQ_ were decreased following treatment with 40, 80, 160 μM artemisinin [Fig. 3]. The lower NPQ in Arabidopsis following artemisinin exposure than the control suggested that artemisinin decreased the non-photochemical quenching capacity termed as dissipating excess excitation energy absorbed by PSII as heat. Localization of NPQ in the antenna system is an efficient means of protective reactions from overstimulation that could result in the formation of reactive oxygen species (ROS) [47]. The increase in Φ_NO_ had already commenced after day 1 and could indicate not only that the plant is under adverse stress conditions, but also that treated plants cannot cope with the stress and dissipate the excess energy into heat through a controlled process. Therefore, lower NPQ and Φ_NPQ_ associated with a lower Chl *a*/Chl *b* contents in Arabidopsis plants probably indicates that artemisinin could function in damaging photosynthesis apparatus through destroying photoprotective thermal energy dissipation mechanism and enhancing ROS generation in stress plants. NPQ is a composite of three different components, each one characterized by a peculiar kinetic behaviour. Artemisinin lowered *q*_N_, Φ_II_ and apparent ETR values as compared to control. The Φ_NPQ_, *q*_N_ *q*_P_ and *q*_L_ were decreased following treatment with artemisinin (Fig. 4).

Malondialdehyde (MDA) is associated with the peroxidation of polyunsaturated fatty acids in the membrane and, consequently, with cellular integrity [48]. Damage to cell membrane indicated by higher concentrations of MDA content that was observed in artemisinin induced stressed Arabidopsis plants when compared with untreated control. Lipid peroxidation indicates oxidative tissue damage by hydrogen peroxide, superoxide, and hydroxyl radicals resulting in structural alteration of membranes with the release of cell and organelle content, loss of essential fatty acids, and formation of cytosolic aldehyde and peroxide products. Artemisinin stress caused an increase in RO of Arabidopsis at all concentrations. ROS species react with lipids and lead to formation of highly active peroxy radical, which in turn start a chain propagation reaction. Root oxidizability helps plant roots to avoid the uptake of toxic materials and provides protection [49] by measuring the oxygen diffusing from the roots into the surrounding environment due to the oxidation of peroxidase; thus, increased RO indicates the enhanced oxidizing ability of peroxidase, as observed in this study. The enhanced RO indicates an increase in respiratory activity, which correlates to enhanced ROS generation. Various researchers also suggest that ROS act as signalling molecules in plants during defence responses, stress responses and programmed cell death [49]. Previously, we found that allelochemicals benzoxazolin-2[3*H*]-one and cinnamic acid decreased the efficiency of photosystem II photochemistry [F_v_/F_m_] and photochemical fluorescence yield (ΦPSII) in *Lolium perenne*, *Dactylis glomerata* and *Rumex acetosa* leaves [45]. However, there is no previous study conducted to check the effect of artemisinin on crops/weeds PSII photochemistry, chlorophyll fluorescence quenching and photon energy dissipation.

Changes in photochemical efficiency, C assimilation and respiration in response to environmental stresses are common in plants; they reflect metabolic adjustments, which include changes in C allocation and N/C balance [50]. In this study, root-respiration (RO) rate increased while C assimilation rate decreased at higher artemisinin concentrations in treated Arabidopsis plant (Table 2). C consumption through root respiration may cause C starvation when C assimilation is inhibited, and may eventually lead to root death under higher artemisinin stress. Leaf nitrogen contents and uptake are costly in terms of energy supply [51]. Increasing artemisinin concentrations increased the root respiration in Arabidopsis that lead to the reduction in leaf nitrogen and carbon contents. The reduction in N is the first sign of senescence due to inhibition in RuBisCO [ribulose-1,5-bisphosphate carboxylase oxygenase] synthesis that contributes to 15–37% in leaf N content [52]. The Ca^2+^ and Mg^2+^ content in Arabidopsis leaves fell progressively following artemisinin treatment as compared to control (Fig. 7). Knowing the nutrient status of the plant could provide valuable information for the interpretation of the phytotoxic effects, especially if there are changes in elements that could influence photosynthetic activity (Fe^2+^, Cu^2+^, etc.) or when the changes affect hormones transport and action (e.g., Ca^2+^ or Zn^2+^). It was found that calcium deficiency can reduce root growth and induce early senescence in the plants [53].

Artemisinin decreased leaf protein contents in *A*. *thaliana* at all concentrations tested (Fig. 8). Early reduction in protein content becomes especially interesting because one of the first effects observed in senescent leaves is the reduction in protein content and, in particular, of thylakoid membrane proteins responsible for the stability of the antenna complex [54]. The highest reduction was obtained following exposure of Arabidopsis to 160 μM artemisinin than control. The change in leaf protein contents in Arabidopsis suggests that protein synthesis or proteolysis is affected by artemisinin treatment. Several reports of alteration of protein synthesis or degradation of protein in Arabidopsis and in other plant species in response to different allelochemicals support our results [45, 55].

Stable carbon isotope composition analysis of leaves is a potential source of information about carbon use properties that could be compared among many plants, weeds, trees, grasses, and within remote forests. The potential changes of isotopic signatures in plant dry matter are of relevance for the use of ^13^C in plant ecophysiological studies. The δ^13^C data are used to study plant water use efficiency, respiration and secondary fractionation processes [56]. There are only few reports in the literature on the carbon isotope composition of CO_2_ respired by plants after allelochemical stress treatment [57]. In our study, the δ^13^C values were less negative following treatment with 40, 80 and 160 μM artemisinin as compared to control [Fig. 9]. At 40, 80 μM artemisinin decreased the Δ^13^C in *A*. *thaliana* leaves as compared to control. Carbon isotope discrimination was reduced following treatment with BOA and cinnamic acid [CA] at 1.5 mM in *Lolium perenne*; *Dactylis glomerata* and *Rumex acetosa* [58]. Barkosky et al. [59], concluded that dried leaf tissue from Leafy spurge (*Euphorbia sula*) treated with 0.25 mM caffeic acid had a less negative δ^13^C compared to controls, indicating less discrimination against Δ^13^C in these plants. Artemisinin (40, 80 and 160 μM) also decreased the ratio of intercellular CO_2_ from inside to outside environment [*c*i/*c*a] when compared with the control in *A*. *thaliana* (Fig. 10). Other researchers have reported that BOA also affect the ratio of intercellular to air CO_2_ concentrations [*c*i/*c*a] in *Lactuca sativa* that was less [0.66] as compared to control [0.69] when treated with 1.0 mM BOA [60]. Similarly, Hussain et al. [58] found that, BOA at 1.5 mM decreased the ratio of CO_2_ concentrations from intercellular to ambient in *L*. *perenne*, *D*. *glomerata* and *R*. *acetosa* relative to control.

## Conclusions

In summary, alterations in Chl *a* and *b* was affected in *Arabidopsis* plants following exposure to artemisinin that indicate not only acclimatory adjustment of pigment composition and photoprotection in leaves, but also escape from oxidative stress. These results demonstrate the occurrence of allelopathy as a mechanism of interference under controlled conditions, but these responses may differ in complex and under the natural field settings. The low fresh biomass, decreased efficiency of photosystem II photochemistry, reduced Δ^13^C, less leaf mineral nutrients, and higher lipid peroxidation attributes to the poor tolerance of Arabidopsis against the artemisinin. The artemisinin produced by *A*. *annua* may be released into the soil either via dead plant material, leaching through rainfall, or the incorporation of plant parts left on the soil after harvest. The artemisinin released into the surrounding environment can cause alteration in growth, biochemical and ecophysiological attributes of neighbouring plants and weeds.

## Supporting Information

S1 File Click here for additional data file. (ZIP)
